# Health-Promoting Nature Access for People with Mobility Impairments: A Systematic Review

**DOI:** 10.3390/ijerph14070703

**Published:** 2017-06-29

**Authors:** Gaochao Zhang, Dorthe V. Poulsen, Victoria L. Lygum, Sus S. Corazon, Marie C. Gramkow, Ulrika K. Stigsdotter

**Affiliations:** Department of Geosciences and Natural Resource Management, University of Copenhagen, Rolighedsvej 23, 1958 Frederiksberg C, Denmark; dvp@ign.ku.dk (D.V.P.); vic@ign.ku.dk (V.L.L.); suoe@ign.ku.dk (S.S.C.); mcg@ign.ku.dk (M.C.G.); uks@ign.ku.dk (U.K.S.)

**Keywords:** accessibility, barriers, disabilities, green spaces, health benefits, health design, nature-related activities

## Abstract

This study systematically evaluated the scientific evidence for health benefits of natural environments for people with mobility impairments. Literature searches based on five categories of terms—target group, nature type, health-related impacts, nature-related activities and accessibility issues—were conducted in four databases (Web of Science, Scopus, CAB ABSTRACT and Medline). Twenty-seven articles from 4196 hits were included in the systematic reviews. We concluded that people with mobility disabilities could gain different health benefits, including physical health benefits, mental health benefits and social health benefits from nature in different kinds of nature contacts ranging from passive contact, active involvement to rehabilitative interventions. Several issues related to the accessibility and use of nature for people with mobility impairments need attention from professionals such as landscape architects, rehabilitative therapists, caregivers and policy makers. The overall quality of methodology of the included studies is not high based on assessment of the Mixed Methods Appraisal Tool (MMAT). Moreover, more randomized controlled trials and longitudinal studies that focus specifically on evidence-based health design of nature for people with mobility impairments in the future are needed.

## 1. Introduction

Over the last decades, research on the association between human health and natural environments has developed into an interdisciplinary research field, which is being carried out in many parts of the world, for example in northern America, Europe, Asia and Australia [1,2]. Even though this research has not yet determined causality, it can be said that the collective amount of research evidence confirms that both visual and physical contact with natural environments is beneficial to human health. According to research, natural environments have a positive impact on human health by reducing the time it takes to recover from stressful events [3], reducing mental fatigue [4], increasing social ties [5] and encouraging people to engage in more physical activities [6]. Current research also suggests that there are synergies between human health and natural environments based on, for example, demonstration of the greater health effects of being physically active in a natural environment compared with being physically active indoors [7].

People with disabilities constitute a large proportion of users of natural environments. As a group, they are subject to increasing attention worldwide with regard to improving their access to different environments. The United Nation’s (UN) Convention on the Rights of Persons with Disabilities, which was adopted in 2006, currently (June 2017) has approximately 174 signatories and parties, including nation states and the European Union [8]. The parties must promote, protect and ensure the human rights of persons with disabilities. According to the World Health Organization (WHO), more than one billion people (approx. 15% of the world’s population) are estimated to have some form of disability [9]. This number is expected to increase due to ageing populations and increases in chronic health conditions (ibid.).

Disability is defined by the International Classification of Functioning, Disability and Health (ICF) as “an umbrella term for impairments, activity limitations and participation restrictions” [10]. A disability is not just a health condition; it is the complex interaction between the individual, and societal and environmental factors (ibid.). Individuals with physical impairments generally face greater mental and physical health challenges compared to the general population [11,12]. A recent Danish nationwide study shows that the respondents’ profile in regard to stress, depression, obesity, social network and health-related quality of life is related to the severity of their mobility impairment [13]. Nevertheless, health promotion and prevention activities seldom target people with disabilities and, due to various barriers from themselves, physical environments and surrounding people, health-care services are less accessible to people with disabilities, causing health disparities.

Despite the increased attention to the positive health benefits of natural environments and the awareness of the UN Convention, people with disabilities have largely been unrecognized within this field of research, and to our knowledge, no systematic review of the body of evidence of the health benefits of nature for people with mobility impairments exists. The objective of present systematic review is to systematically evaluate the scientific evidence for health benefits of the use and design of natural environments for people with mobility impairments. Specifically, this systematic review examines:

- What types of nature interventions and activities for people with mobility impairments are found in research studies?
- What characterizes (diagnosis; mobility impairment; gender and age) the different groups of people participating in the studies.
- What health outcomes are addressed in the studies?
- What is the quality of the studies, based on qualitative, quantitative, and mixed method designs?
- What accessibility issues are perceived by the target group during nature interventions?

## 2. Methods

### 2.1. Literature Search Strategies

The literature search started with an initial search using Google to get an overview of the key words for the following systematic literature search. We identified a number of search terms that were used in the following systematic search in different databases. These terms (Table 1) were divided into five different categories: target group, nature type, nature-related activities, health impacts and accessibility issues. In order to catch as many relevant studies as possible, terms in the three categories “target group”, “activities” and “health impacts” ranged from general terms and specific terms used in the research. The search terms for the categories “nature-related activities” and “nature” were used to obtain a more comprehensive result based on an understanding of nature as having an impact on individuals who spend time there by providing sufficient places for individuals to carry out different kinds of physical activities.

Next, systematic searches were carried out in four databases (Web of Science, Scopus, CAB ABSTRACTS, Medline). To maintain high relevance for our focus, the search in each database was based on a combination of terms from four categories. The terms “target group” and “nature”, which are essential for this systematic search, were included in every search. The database searches were carried out without year restriction and research field restriction. Terms that belong to the same category were combined with “OR”, and terms that belong to different categories were combined with “AND”. Searches in Web of Science were conducted in “topic”, searches in Scopus were in “Title-abstract-keywords”, and searches in CAB ABSTRACTS and Medline (these two database use the same search system) were in “title” and “abstracts”. As a consequence of the inherent difficulties of exploring such a heterogeneous subject, we widened the scope outside the protocol from time to time by using snowballing in order to obtain as much adequate information as possible.

### 2.2. Selection Criteria

No restriction was applied for study types. The titles and abstracts of the hits were assessed for initial eligibility. The studies whose themes were potentially related to our objectives were selected for the full text screen. The full texts of the selected studies were screened by the author based on the criteria described below:

*Context*: Different kinds of nature where natural elements are more prevalent than man-built features. Studies in which outdoor environments are streets, outdoor facilities in communities and other kinds of man-made outdoor environments which are more of built-environment than nature were excluded.

*Participants*: Individuals of all ages with mobility impairments were eligible as our target group. Mobility impairments are very common among the elderly. Therefore studies whose target groups are elderly people and that focus on mobility impairments were also considered as eligible. Studies that focus on more than one aspect relating to the elderly were also considered as qualified if the mobility impairment problem was one of the most important foci of the study, and if the study included important findings which fall into the scope of our research points. Decision to include studies with other target groups and other levels of disability was based on the content of the study.

*Activities*: Activities must be nature-related. In other words, activities from the included study must be carried out within the context of our definition of nature, for example, nature-based recreations and habitual nature-related activities. Moreover, rehabilitation activities carried out in natural environments were also accepted when nature provided a unique impact that could not be provided by other environments and facilities.

*Outcomes*: Health (physical health, mental health and social health) impact measured objectively or subjectively. Also impacts that contribute to a healthier lifestyle were considered to be health benefits. The measurement could vary from medical indicators to self-reported outcomes.

*Accessibility issues*: We only considered accessibility issues related to the outdoor nature that resulted in a better use of nature with scientific and reasonable solutions. Barriers found in indoor locations, in human-built streets and in transportation were not included in our scope.

### 2.3. Data Extraction

The main characteristics (study type, main characteristics about the participants, type of nature and nature-related activities and main outcome reported) were extracted from the included studies. The outcomes are presented with a summary by the authors because the included studies fall into a wide range of research fields.

### 2.4. Quality Assessment

This review study includes quantitative, qualitative and mixed-method studies. The Mixed Method Appraisal Tool (MMAT) version 2011, a tool designed for the assessment of the quality of studies for systematic reviews including qualitative, quantitative and mix-method studies, was applied in the assessment of the quality of the methodologies used in the included studies [14]. The MMAT checklist has two initial screening questions and 19 components corresponding to qualitative research, randomized controlled trial studies, non-randomized studies, quantitative descriptive studies and mixed methods studies. It uses a scoring metric whereby each study is scored between 1 and 4, where 1 is the lowest and 4 is the highest quality. All included studies are scored based on whether they meet the specific criteria regarding the methodology related to the specific type of study. Criteria concern, for example, data collection, data analysis, whether there is a complete set of outcome data, and consideration of the influence of the environment. Each domain has criteria with which to evaluate the quality that can be presented with the descriptors of *, **, *** and ****.

## 3. Results

### 3.1. Study Identificaiton and Selection

**Figure 1:**
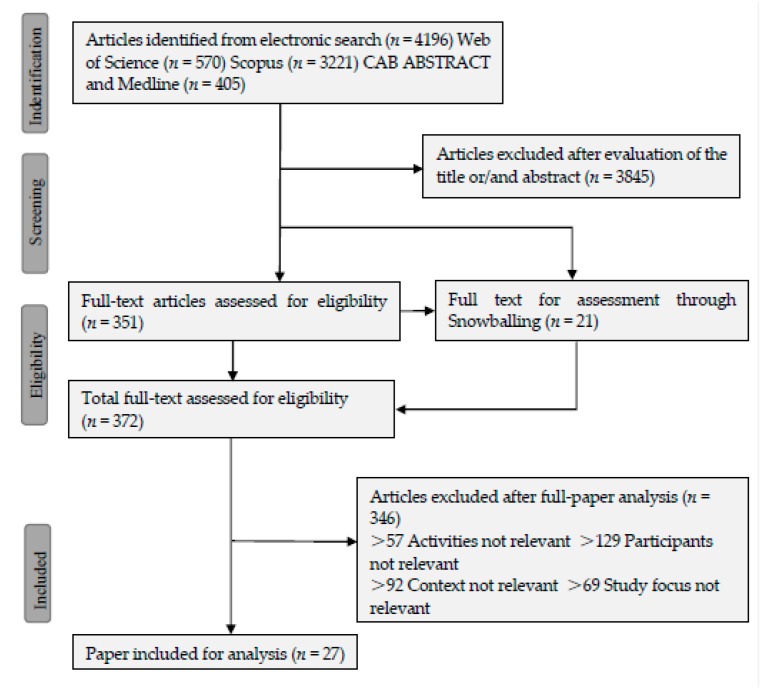
Flow diagram for the identification of eligible studies (Only one reason is listed per excluded study, but in many cases, there was more than one reason for exclusion).

We got a total of 4196 hits (Web of Science 570; Scopus 3221; CAB ABSTRACTS and Medline 405). Due to the reasons stated above and because we aimed at high sensitivity we could not adhere solely to our predefined search protocol, but were forced to use extended sources to obtain as much information as possible. This resulted in rather low precision in our first yield, as is reflected by the number of the hits and selected literatures after the first round of selection where 351 literatures were selected for the second round of selection. For all the studies described in this review, as a minimum, the abstracts were read. In addition to the papers from snowballing, the full texts of the articles whose abstracts qualified were read for selection based on the outlined criteria. This process resulted in the final selection of 27 articles (Figure 1).

### 3.2. Study Characteristics

The number of the included studies focusing on different mobility impairments varies (Table 2). During our study, we found that there is much research about the elderly. We have ruled out the elderly studies that do not focus on mobility impairments. Despite this round of exclusion, the majority of studies included focus on elderly still number the most (8 out of 27). The second largest group of studies included deal with Parkinson’s disease (6 out of 27) which is followed by spinal cord injury (3 out of 27).

The review includes 14 quantitative studies, 10 qualitative studies and three mixed methods studies. Specifically, the review includes cross-sectional analytical studies (5), case series (3), randomized controlled trials (3), quantitative descriptive study (2), non-randomized controlled trial (1), phenomenology (5), qualitative description (3), case study (2), triangulation design (2) and embedded design (1). No longitudinal study was included (Table 3).

In general, the methodology quality of the included studies is not high. Specifically, only four studies were ranked with ****, which indicates a high quality about the methodology. Nine of the included studies were ranked with ***. The remaining 14 included studies were ranked with ** or *, which indicate a poor quality of the methodology.

Overall patterns about the participants, context and activities for the excluded studies that surfaced after reading the full text indicated current research interests from the relevant research fields. Many studies about the elderly were excluded because they failed to define the mobility status of the participants. In addition, a number of studies were excluded because they did not distinguish between different types of disability. With regard to context, many studies used outdoor space as an umbrella word that covers a more general category of the outdoor environments such as streets and other outdoor facilities that are human-built outdoor environments. This reflects a growing research interest in people with impairments from relevant fields, such as city planning and architecture. Regarding the activities, outdoor activities in some studies were not nature-related, for example, some studies from sport science studied sports in outdoor sport fields. Furthermore, some outdoor activities are more general, for example, going out for shopping purposes or similar. A number of excluded studies about outdoor recreational activities focusing on people with mobility impairments were about general outdoor recreational activities, such as visiting historical sites. Most of these studies were about recreation or tourism.

### 3.3. Nature-Related Activities

The nature-related activities from the included studies were divided into three categories:

• Passive involvement

Passive involvement includes watching nature and being in nature to observe nature and relax. Two studies highlighted the well-being benefits of watching nature from the window [25,38]. In the study by Kearney et al., the authors pointed out that when the study participants watched from the window, they most frequently expressed pleasure when looking at views of plants and birds. In contrast, built-environments and a lack of vegetation were most frequently mentioned as a view they disliked [38]. Staying in nature is also a common type of nature contact. When staying in nature, people with mobility impairments could observe the natural plants and animals [28,30], and enjoyed different kinds of sensory stimuli [24]. One study stated that the target group enjoyed the pleasure from stimulus of different senses, for example the fragrance of the plants from nature [26].

• Active interactions

A wide range of active nature-related activities is available to people with mobility impairments, ranging from normal walking activities to challenging outdoor activities. Mobility impairments do not necessarily preclude people with mobility impairments from participating in the outdoor physical activities. Walking [22,23,26], Nordic walking [19,21] and gardening [25,26] are the most common activities in the included studies. Activities like canoeing [35], kayaking [17] and skiing [32] are also reported in the included studies. Finally, there are recreational nature-related activities like fishing. Freudenberg made a cross-sectional analytical study about recreational fishing and found that anglers with disabilities participated in fishing quite often [37]. There are also some nature-related physical activities that are quite physically challenging for the target group. People with limb deficiencies were shown to also master skiing with sufficient practice [32]. In another study, SCI patients enjoyed sea kayaking because it offered them the chance to be free of their wheelchair and act like others [17].

• Rehabilitative interventions

Some research organized rehabilitative activities in nature for the improvement of certain indicators of the physical impairments of Parkinson’s disease [21]. In addition, some nature-based activities were organized by occupational therapists, with a view to achieving a therapeutic objective through the experiences. The nature-related activities from three of the included studies can be labelled as Outdoor Experiential Therapy (OET) programmes [16,22,23]. This kind of programme uses the outdoor environment as a therapeutic setting and incorporates a variety of therapeutic modalities [42]. Usually there are one to several active nature-related activities included in one certain rehabilitative intervention. For example, the Therapeutic Recreation Cottage Program has evolved to be a programme that consists of a variety of outdoor recreation activities such as hand cycling and canoeing [16]. This kind of therapy is participant-centred and provides the patients with “direct experiences” by allowing them to take action themselves rather than being passive observers.

### 3.4. Health Impacts from Nature and Nature-Related Activities

Overall, nature and nature-related activities provide a range of health benefits. In Table 4, the health benefits are divided into three categories, physical health benefits, mental health benefits and social health benefits.

#### 3.4.1. Physical Health Benefits

Two studies pointed out that participating in nature-related activities improved the target group’s overall life skills as well as the skills needed to participate in the activities in which they participated [32,35]. SCI who participated in sea kayaking experienced improved strength and stamina [17]. Nature-related activities such as canoeing are believed to be an ideal medium for people with disabilities to gain and use different skills in the natural complex functional setting [35].

In five studies, Parkinson’s disease (PD) patients improved their mobility measured as a change in their symptom indicators [18,19,21,22,39], and these benefits were usually more significant than rehabilitation activities carried out in non-nature environments [18,21]. In addition to improvement with regard to mobility, PD patients also experienced other physical health benefits such as improved cardio-respiratory capacity with significantly lower and stable blood pressure and heart rate in exercise [21]. Most often, these benefits appeared immediately after the activity and many of benefits transferred into lasting effects in the patients’ subsequent daily life [19,22,39]. Some researchers believe that long-term health benefits may be due to the lifestyle change that led the patients to be more physically active [19], or that they were the result of the patients’ improved self-confidence and self-motivation after having walked in physically taxing mountain environments [22].

#### 3.4.2. Mental Health Benefits

Participants in three studies expressed better mood and relaxation following nature and nature-related activities [17,26,41]. Rodiek found that elderly people with different mobility levels were less stressed after spending some time in an outdoor natural garden environment than the control group who spent the same amount of time in an indoor non-garden environment as measured by the reduction of cortisol [30]. Some of the participants expressed that they experienced a positive relationship between spiritual inspiration and connection [26], relief of pain and other types of distress [28], improved cognition [21], sense of freedom and renewal [24], and time spent in nature.

More mental health benefits were related to the improvement of personality. The target group gained positive affect and better self-efficacy [16], self-improvement [37] as well as better self-confidence [39] that they could transfer to their everyday life from different nature-related activities. In three studies, the participants frequently described that they felt enslaved by illness, but realized after participation in the activities that they could in fact do things, even with their disabilities [17,23,32]. SCI patients who participated in sea kayaking mentioned that the feeling of being free of their wheelchair when in the sea gave them a feeling of equality. They thought that this experience made them better at coping with difficult periods, that it was a good way of defusing stress and led to better self-confidence and self-esteem [17]. Adolescents who participated in a skiing programme achieved better self-esteem by acknowledging what they could do despite their disabilities [32].

#### 3.4.3. Social Health Impacts

The social benefits associated with recreational fishing were found to be more significantly perceived by anglers with disabilities than by anglers with no disabilities [37]. These benefits were believed to be associated with the disabilities status rather than other demographic features such as age [37]. Disabled participants in a wilderness canoeing trip gained significant improvement in social health, including better tolerance of others, were more comfortable when meeting new people, and increased their involvement in society [35]. Gardening enhanced social interaction, communication and support from different groups [41]. In a case study carried out in Norway targeting an amputee, the participant expressed that he quite enjoyed socializing with people after he had regained contact with nature upon returning home following his stay in hospital and a nursing home. Furthermore, he was convinced that many people from the institutions and care homes were “dull” as a result of the lack of physical activities and fresh air [33].

Finally, when performing challenging outdoor activities such as skiing, participants may experience enhanced social health as a consequence of the support they get from families and friends [32]. Also it is believed that nature is an ideal place for social interactions and engagement [26].

### 3.5. Mechanism of the Health Impacts

Most of the studies included in the review do not explore directly how health benefits are generated. However, some hypotheses are put forward. Rodiek believed that the nature perception that includes changing and unpredictable smells, sounds and light is a unified and multisensory experience that plays an important role in the health effects [30]. About the nature-related activities, although indoor or urban alternatives for achieving similar goals may exist, nature can provide a number of additional therapeutic benefits that are thought to stem from the nature itself [43].

There is also analysis based on the theory of phenomenology. Here, the target group is believed to use more physical and psychological energy when performing activities that are easy for the able-bodied population. This leads to frequent reminders of their own impairments, resulting in the participants feeling that their life turns from “being towards the world” to “being towards the body” [23]. When they succeed at performing activities that are not common in daily life, participants feel that their impairment-related boundaries are stretched in a positive direction, enhancing their sense of fitness, their ability to cope with daily challenges, and their harmony between life and mind [23]. This view tallies with the experiences of the participants in three studies, who expressed that they got new perspectives from being in nature environments that are very different from their daily living environments [17,23,32].

For some activities that take place in a context requiring an involvement of different human systems, health effects may fall into the category of occupational adaption. Occupational adaption premises that humans need to respond to different occupational challenges to promote their health and well-being, and posits that these challenges take place in a context of a combination of physical, social and cultural systems [32,44].

Also, there is a view that nature reduces some illness-related symptoms by acting as a space that conveys no danger compared to the human-built environment. This is based on the result that freeze of gait (FOG) symptoms in PD participants are only absent in natural environments where there are no human-built structures [20].

### 3.6. Preferences

The perception about nature varied between individuals with different backgrounds. However, a common understanding was that a good natural environment for the target group should be include better safety [26,40], accessibility [26,31,36], walkability and should not be interrupted by traffic [26]. It is also important to make more comfortable resting facilities [40] and better open views [31]. However, in addition to considering special requirements for target users, it is also essential that environments are aesthetically pleasing [25,40].

Natural features are valued most by the target group [17,24,25,26,31,36,38]. Some individuals from the target group even saw natural elements such as fresh air and vegetation as a motivating factor for outdoor activities [26,38], and in the eyes of some participants, they are even the reason to “get out the door” [26]. Different sensory pleasures from water, plants, flowers and wildlife are also important factors that contribute to participants’ enjoyment in nature [24,25,40].

### 3.7. Barriers for Nature and Nature-Related Activities

Physical barriers come from many ways. Significant slopes, sudden height differences and insufficient width of a walkway [24,41] are common examples of barriers for individuals with a physical impairment. The materials a path is made of may also pose a serious barrier [24,38,41]. For example, high contrast paving patterns may confuse some users who read the dark pavers as voids and may resist using the pathway [38]. Poor and improper ground surface like ground covered with stones and bumps is not good for users of walking assistance tools [24,38]. To deal with some of these barriers, support facilities such as handrails and resting facilities can be essential because they can help users to maintain their balance [24,38]. Spaces should have legibility of circulation and clear visual access to entries and exits to reduce the potential danger arising from poor mental capacity [38]. Protection in the event of bad weather is also essential as participants frequently mention lack of protection as a barrier [38]. Accessibility is important does not mean some characteristics of nature should be sacrificed for it. Some studies found that participants with disabilities do not want the wildness of natural environments altered in order to make the environment more accessible to them [39]. Providing information about the level of access available in wild, natural areas may be a good way to achieve this balance [39].

Individuals with mobility impairments encounter other barriers than physical barriers. A large ratio of participants stated that insufficient assistance from others held them back from going outside [28,38,45]. The attitudes from the surroundings also influences their participation in nature [17,25]. SCI patients who participate in the sea kayaking mentioned that they hope there could be more awareness about activities that persons with SCI can do, and they expressed their desire for more support to be given to outdoor activities such as sea kayaking [17]. Furthermore, some barriers were lodged in their mind beforehand. For example, one study showed that participating in a programme consisting of different outdoor activities decreased the barriers perceived in the minds of individuals with mobility impairments [16]. An interview study pointed out that familiarity with the area is very important, because the target group had more anxiety about dangers such as getting lost than others [24].

## 4. Discussion

Overall, based on the evidences from the included studies we know that nature does have positive health impacts for people with mobility impairments. At the same time, the included studies provide some information which could be beneficial to the practices of rehabilitation and landscape architecture and researches in the future. However, the knowledge about evidence-based health design is far from adequate.

### 4.1. Health-Promoting Nature Seen from the Perspective of People with Mobility Impairments

**Figure 2:**
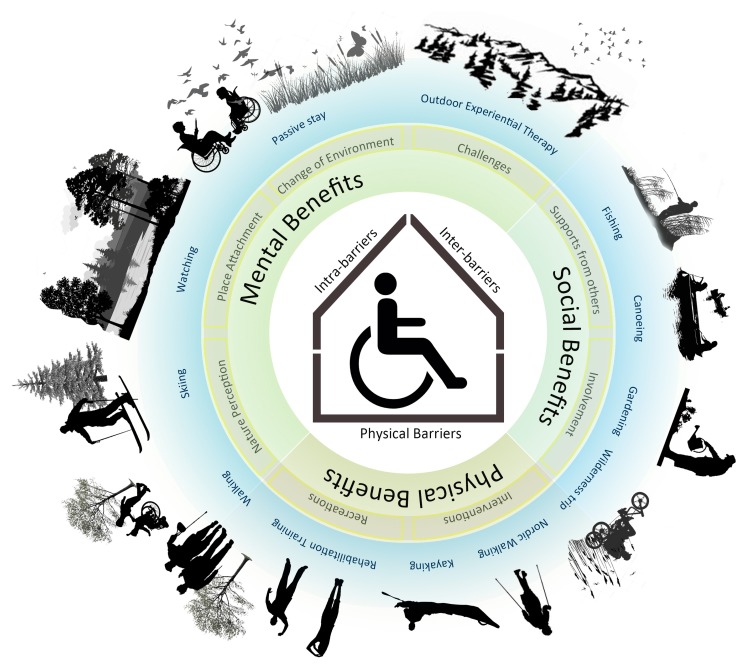
A summary of the findings about health-promoting nature for people with mobility impairments. This figure shows three categories of health benefits from nature, the main ways in which nature implements these health benefits and the three categories of barriers for people with mobility impairments as summarized from the included studies of this systematic review.

From the included studies, we gained an overall picture of the health benefits provided by nature for people with mobility impairments (Figure 2). Nature attracts them and offers them an ideal place to go to, to perform physical activities that can contribute to their physical health. Moreover, for some rehabilitation activities, the natural features of nature are natural training spaces in which rehabilitation training for patients of Parkinson’s disease can be carried out.

Of the three categories of health benefits, mental health benefits are the most commonly mentioned. Mental benefits such as stress relief, improvement of cognition and relaxation are at the same level as for the able-bodied population. Some unique health benefits are improvements that positively affect the individual’s personality. When talking about the health benefits of nature and nature-related activities, the benefits most frequently mentioned by the target groups are improvement of self-confidence, self-esteem and the realization of their capacity. This is not surprising as individuals with physical disabilities usually find it challenging to venture into nature and perform some activities, especially the challenging activities. Sometimes the subconscious assumption, both by the target group and people surrounding them, that they are unable to perform these activities, make them lack belief in their ability to enjoy nature. Once they have experienced nature as enjoyable, just as the able-bodied population does, they gain a more positive view of their abilities and their limitations. This view contributes to a more positive self-identity with regard to coping with possibilities and challenges in the future. Social health benefits may come from the new attitudes towards social involvement and interactions. For example, activities such as trips into the wilderness with a combination of people with disabilities and the able-bodied population were usually considered by people with mobility impairments to lead to a new perspective on their social life. Moreover, nature provides a more supporting space for the target group to communicate with others than their previous common indoor scenes. Based on the research reviewed, we may also deduce that the health benefits of nature for people with mobility impairments seem to have long-term effects. Some of the studies included provided some evidence of the positive long-term impacts [22,39]. At the same time, the change of life style is also frequently mentioned [19,23,26]. It is reasonable to say these changes will contribute to the health of people with mobility impairments as it is without doubt that an active life style is beneficial, whereas inactivity may lead to health problems such as depression.

In general, people with mobility impairments have the intention to enjoy nature if possible. Natural elements such as big trees, wildlife, the sunshine and the fresh air are broadly valued. These elements are believed to bring relaxation as well as connection to the world and past life experiences. Also, enjoyment caused by stimulation of different senses is also important for them when they talk about the contact with nature. Smelling the fragrances of plants and hearing the sounds of the water and birds are frequently mentioned as enjoyable sensations. These sensory experiences are unique experiences that are not possible to get from the indoor environment. As a result, we can reasonably say that when comparing to the indoor environment, nature provides a uniqueness that attracts people with mobility impairments.

Barriers for people with mobility impairments are not limited to physical barriers such as the inaccessibility of paths. Crawford and Godbey identified three classes of constraints for participation in recreational activities: interpersonal, intrapersonal and structural constraints [46]. Intrapersonal constraints involve the individual’s internal psychological processes that affect preferences toward specific activities. In the case of people with impairments, this group is frequently constrained by their physical challenges. As a result, they have a lower expectation about what they can do. Furthermore, their relatively lower self-confidence and sense of security may prevent them from going outside. Interpersonal constraints result from interactions with other individuals. Raising society’s awareness of what people with mobility impairments can actually do may help reduce interpersonal constraints. The support from different groups could help people with mobility impairments to make more nature-related activities into action. When individuals with mobility impairments succeed at an activity this helps reduce their intra-barriers. As for structural constraints for people with mobility impairments, the main issue is physical barriers, for example barriers regarding accessibilities and the physical constraints of certain physical activities. This is understandable based on the limited mobility of the group. Having said that, it must be stressed that even though accessibility is valued by the target group, this does not mean that nature should be as accessible as possible; a sense of wildness and natural elements is highly valued by the group. As a result, when working with the physical barriers, a balance between the wild features of nature and accessibility should be sought. Wilderness should not be traded for accessibility, and accessible nature should be achieved under the premise that the precious essences of nature should be maintained as they are so essential to the nature-related benefits.

### 4.2. Implications for Future Practices and Suggestions for Future Research

It could be beneficial if professions like the therapists and caregivers apply more nature-related rehabilitative activities for people with mobility impairments for the purpose of better health-related quality of life. For example, more rehabilitative physical activities could be carried out in nature environments such as parks and gardens with the supervision of a professional therapist. In addition, organized activities that can link people with disabilities with wilderness should be developed with a health-promoting intention. This may lead to people with mobility disabilities being offered more opportunities to participate in a wider range of activities. More attention should be paid to the green spaces that surround the residences of the target group, such as long-term health care facilities, and community spaces where the target group gathers. With the correct design and management, green spaces haves the potential to be a resource for health benefits by providing enjoyment of nature and by being an ideal place to perform outdoor activities.

Landscape architects should allow the addition of more natural elements during their design process of different green spaces. The included research has shown evidence that the target group prefers more vegetation in outdoor green spaces. Also structures that provide shelter from bad weather should be set with appropriate consideration to the universal design to accommodate the target group’s sensitivity to different kinds of danger. We believe that most physical barriers can be eliminated or alleviated through the use of functional design and management. Furthermore, this design should be based upon evidence from scientific research rather than subjective hypothesis.

Accessibility is an important issue due to the limited mobility of people with mobility impairments. However, this does not mean accessibility should be weighted higher than all the other issues; as stated in several of the studies, the target group expressed their appreciation of the “wild”. To obtain a balance, a solution may be to provide information clearly indicating the accessibility level of the unknown nature and the potential dangers and barriers. This will provide a better overall image of the difficulties of the journey and strength required to complete it. Furthermore, other categories of barriers should be kept in mind. For example, the awareness of the general population should be raised about the ability rather than the disability of the target group. Moreover, caregivers and people around the target group should offer more help to them to experience nature. Furthermore, more policies that encourage social help for people with mobility disabilities to enjoy nature should be discussed by policymakers.

More scientific research about how to make more supporting nature for the purpose of health promotion of people with mobility impairments should be carried out in future. This research should not be limited to the scope of physical accessibility. The current evidence level for the health benefits from nature for people with mobility impairments is not concreted based on the methodology quality assessment and number of included studies. More evidence, especially evidence based on random controlled trials, and evidence focusing on the health benefits of daily use should be provided. Abundant and convincing evidence is the basis for evidence-based health design. Moreover, more research on the barriers of the target group when using nature should be carried out. The main focus of this systematic review is health-promoting nature, and studies whose main focus is on the accessibility issue should be carried out to give us a clear image about the barriers for the target group.

### 4.3. Limitations for This Review Study

The main limitation for the present review was the small number of the included studies with a relatively small sample size. This means that it is not easy to draw generalized conclusions. Furthermore, the quality of the included studies is not even. Some of the studies do not have high reliability. Only three of the 27 included studies are randomized control trials. This may due to the innate difficulty of this kind of study as it is difficult to allocate a convincing control group and control the variables when conducting the study. Also the physical limitations of the target group usually make it difficult to obtain a sufficient number of participants that can ensure a better quality of the study. As for the methodology, the included studies were all identified from a search in scientific databases. There may be a possibility of the omission of some good grey literature. Moreover, all the studies were in English, which may exclude some relevant studies written in other languages. This hindered us from knowing the cultural differences about health-promoting nature for the target group. Finally, it is difficult to obtain an image with in-depth information from different perspectives regarding health-promoting nature in one systematic review study. Therefore, more studies are needed.

## 5. Conclusions

In general, nature and nature-related activities are health promoting for people with mobility impairments. Overall, the health benefits covered physical health benefits, mental health benefits and social health benefits. Among the three identified categories of health benefits, psychological health benefits are supported by the most studies. The various health benefits may come from the natural elements in nature, change of environment and the accomplishment of activities that the target group thought to be beyond their abilities. Our systematic review also indicates that barriers are common when it comes to the usage of nature for people with mobility impairments, and that the barriers are not merely the lack of physical accessibility, but also comprise invisible intrapersonal and interpersonal barriers. People with mobility impairments also expressed clear preferences for natural features such as trees and described feeling pleasure from other senses such as the sounds of water and birds in the studies. However, the available studies do not provide a sufficiently strong basis of evidence. Further research with high quality, especially random controlled trials and longitudinal studies, are needed. These studies should have a clearer focus on the mechanism of the health benefits and demonstrate a closer correlation between the health benefits and the usage issues of nature for people with mobility impairments to provide more practice-applicable evidence. Consequently, more interdisciplinary collaborations focusing on health-promoting nature among the target group, therapists, landscape architects, policy makers etc. should be established to make the health benefits, a key component of nature’s benefits, be offered to people with mobility disabilities for their better health related quality of life.

## Figures and Tables

**Table 1:** Search terms.

Category (CAT)	Target Group	Nature	Nature-Related Activities	Health Impacts	Accessibility Issues
All search words are combined with “OR” within each category	- Walking disabilities - Walking handicaps - Physical disabilities - Physical impairments - Mobility problems/impairments - Elderly - OR some specific targets: - Cerebral palsy - Rheumatism - Muscular dystrophy - Parkinson’s - Spina bifida - Spinal cord injury - Sclerosis - Polio - Arthritis - Osteoporosis - Neuromuscular disorders	- Natural environment - Wilderness - Forest - Wood - Outdoor - Green space - Greenspace - Park - Garden - Open space	- Sport - Motion - Training - Exercise - Workout - Fitness - Adventure - Outdoor recreation - Outdoor activities - Or Specifically - Boating - Fishing - Hiking - Skiing - Swimming - Riding - Walking - Swing - Hand cycling - Wheeling - Kayaking - Gardening - Horticultural activities	- Health Benefits - Health Promotion - Health effect - Health outcome - Health improvement - Wellbeing - Quality of life - Quality of life (QoL) - HRQoL - Healthrelated quality of life - Rehabilitation - OR specifically - Physical performance - Physical wellness - Balance - Mobility function - Physical capacity - Fitness - Endurance - Flexibility - Functional capacity - Strength - Happiness - Restoration - Enjoyment - Spiritual benefits - Satisfaction - Self-esteem - Confidence - Peacefulness - Social connection - Social involvement - Social benefits - Strengthened relationship	- Accessibility - Universal Design - Inclusive Design - Design for all - Barrier free - Accessible - Landscape architecture - Planning

These search terms are basic terms. When carrying out searches in the database, we used several wildcards in accordance with the rules in different databases to help us to get more related hits.

**Table 2:** Mobility impairments type of the participants from the included studies (*n* = 27).

Variables	Number of Included Studies of Certain Mobility Impairments						
Type	SCI	PD	MS	Elderly	Limb Deficiency	Cerebral Palsy	Others/Not Specified
N_1_ + N_2_	3 + 1	6 + 1	0 + 2	8 + 2	2 + 2	1 + 1	7 + 1

N_1_ is the number of studies that only focus on certain kinds of mobility impairments; N_2_ is the number of studies include a certain impairment from studies that focus on different kinds of impairments; SCI—spinal cord injury; PD—Parkinson’s disease; MS—multiple sclerosis.

**Table 3:** Main characteristics and results of the studies.

Author (Year) [Reference]	Country	Research Design	Sample	Mobility Impairments	Nature/Greenspaces	Contact Type	Health Impacts	Other Points	Quality
Botticello (2015) [15]	U.S.	QUAN/Cross-sectional analytic study	503	SCI	Community green spaces	-	More mixed land use and small amounts of green spaces—Poor perceived health	Inaccessible and highly developed environment may exacerbate the deleterious effects of stress	**
Hitzig (2012) [16]	CA	QUAN/Non-RCT	21 (14 EXP; 7 CON)	SCI	Multi-kinds (Land and water)	OET	Self-esteem, affect and self-efficacy ↑	Participants are satisfied with the programme and willing to participate more	***
Taylor (1996) [17]	U.S.	QUAL/Qualitative description	3	SCI	Sea	Active involvement/Kayaking	Relaxation, stress defusion, self-esteem, self-confidence; social interaction; Physical strength, stamina, balance ↑	Nature atmosphere is appreciated; perceived safety; need awareness and support from surroundings	**
Ebersbach (2014) [18]	DE	QUAN/RCT	58	PD	Local parks	Rehabilitative intervention/supervised NW	Immediate and follow-up cognitive aspects of movement preparation ↑	-	**
Frank (2008) [19]	NL	QUAN/Self-controlled Case series	19	PD	City Park	Active interaction/NW	Walking speed, Timed up and go test (TUG) test, timed walking distance and QOL↑	More active life style in long-term future	***
Ottosson (2015) [20]	SE	MM/Triangulation Design	5	PD	Natural environment as compared to built-environment; Site-Alnarp rehabilitation garden	-	Nature induces less freezing of gait than built environment; Better physical performance in nature	The visual environment should try to convey signal of “no danger“ to reduce the FOG.	** (QUAL **; QUAN **)
Reuter (2011) [21]	DE	QUAN/RCT	90	PD	Park and forest	Active involvement/Nordic walking and walking	HRQoL, cognitive function, postural instability, mobility, cardio-respiratory capacity ↑	NW is more preferred than walking and indoor training. More active future life for NW participants.	****
Sunvisson (1997) [22]	SE	QUAN/Self-controlled Case series	12	PD	Mountain	OET (1 week walking a year in 3 consecutive years)	Immediate overall motor performance and coordination capacity simultaneous integration ↑ Additional improvement in follow-up	More active outdoor life after participation; a combination of training and social interaction with counterpart contributed to the benefits.	***
Sunvisson (2000) [23]	SE	QUAL/Phenomenology	11	PD	Mountain	OET (1 week walking a year for 2 consecutive years)	Feeling of capability, social interaction, self-confidence, self-esteem and positive life attitude ↑	Social relationships and challenges in the wildness trip make them feel less trapped by daily negative experiences	***
Bengtsson (2013) [24]	SE	QUAL/Phenomenology	12 patients + 7 next of kin	Elderly residents in nursing homes with limited mobility	Surrounding nature of nursing homes	Passive contact mainly	Sensual pleasure, connection to the past; social involvement ↑	Fresh air, light and greenery + Different senses like the sound of water and birds and the natural fragrance are appreciated; Accessibility, safety, familiarity and the rights of choice are important.	****
Brascamp (2004) [25]	NZ	MM/Embedded design	61	Elderly typically with physical limitations	Green environment near retirement homes	Passive involvement and active interaction (Gardening)	Relaxation, perceived wellbeing and satisfaction ↑	Passive involvement is perceived to contribute more to wellbeing. Staff encouragement contributes to outdoor enjoyment.	* (QUAL *; QUAN **)
Finlay (2015) [26]	U.S.	QUAL/Qualitative description	141	Elderly with limited mobility	Blue and green spaces	Passive contact: Nature exposure; Active interaction: walking, gardening and hiking, etc.	Physically active lifestyle, physical strength; spiritual peace and connectedness, rejuvenation; social interaction, social integration, etc. ↑	Natural sights, sounds and smells are valued; waterscapes are highly preferred; Rest spots and shelters could enhance usage. Distance, Slippery and uneven surfaces (walkability) induce fears.	****
Gong (2014) [27]	U.K.	QUAN/Cross-sectional analytical study	1010	Elderly with different level of lower extremity	400 m radius outdoor environment around home	-	More green space—higher physical activities	Homogeneous vegetation has positive relationship with physical activities	***
Rappe (2006) [28]	FIN	QUAN/Cross-sectional analytic study	45	Elderly women with different level of mobility	Outdoor green environments of long-term care	Passive involvement/visit or watching	Pain reduction, tranquility; self-related health ↑	Restriction of visit: Lack of assistance, bad weather, steep or uneven paths, poor health, door and doorstep; Trees, fragrant flowers and birds are appreciated	***
Rappe (2005) [29]	FIN	QUAN/Cross-sectional analytic study	26	Elderly with different level of mobility	Gardens of long-term care	Garden visiting	Mood, feeling of recovery, sleep quality, feeling of balance, concentration, pain relief ↑	Hindrances: Lack of assistance > bad weather circumstances > steep and uneven paths; Natural elements such as plants, smells, fresh air, activities and animals are main motivations	**
Rodiek (2002) [30]	U.S.	QUAN/RCT	16	Elderly with different level of mobility	Outdoor horticulture garden	Passive involvement/Observing	Mood and anxiety level (improved but not significant); Stress level ↓	-	**
Rodiek (2005) [31]	U.S.	QUAN/Case series	133	Assisted living elderly with different level of mobility	Nature surrounding the assisted living facilities	-	-	Places to walk, trees, resting spaces and different views are preferred	**
Pasek (1995) [32]	U.S.	QUAL/Phenomenology	14	Limb deficiencies	National sport centre for the disabled in Winter Park, Colorado	Active interaction/Skiing	Activity performance; self-satisfaction, self-esteem, satisfaction with others ↑	Mastery of skills and social interaction may result in better self-esteem	***
Ramsland (2015) [33]	IT	QUAL/Case-study	1	Elderly amputee	Nature around his home in Ålesund	Mainly passive contact	Perceived mental and physical health, mood, positive attitude; social health ↑ stress ↓	Accessible paths make it possible to go out; Natural elements like the sunshine, air, scents, bird sounds are appreciated.	**
Dawson (2009) [34]	U.S.	QUAL/Phenomenology	27	Cerebral palsy	Outdoor centre with universal accessibility	Summer camping including different kinds of nature-related activities	Social involvement and interaction; self-esteem, self-confidence ↑	The interpersonal relationships and atmosphere are believed to contribute to the benefits	**
Anderson (1997) [35]	U.S.	MM/Triangulation design	12 with disability + 14 without disability	Different kinds of disabilities	Wilderness	Active interaction/Integrated outdoor adventure program including canoeing as a main part	Social integration, interpersonal relations; relaxation, personal growth, positive attitude toward disabilities, positive lifestyle; Skill development ↑	Integrated participation with people without disabilities are helpful in different aspects	** (QUAL *** QUAN **)
Brown (1999) [36]	U.S.	QUAN/Descriptive study	197 (116 with mobility limitation + 81 companions)	People with mobility limitations without classified diagnosis	Scenes of Parks	-	-	Forests are preferred to open fields especially the ones with paths; Feathers that could make them feel confident and comfortable are also important.	***
Freudenberg (2009) [37]	DE	QUAN/Cross-sectional analytic study	775 (345 with physical disabilities and 428 without)	Varied kinds of physical disabilities and not specifically stated	Fishing sites in wilderness	Active interaction/Recreational fishing	Social interaction benefits, benefits for self-improvement, nature and relaxation-related benefits and challenge-related benefits ↑; Social benefits and benefits for personal growth more significant than people without disabilities	People with disabilities fish more frequently than people without disabilities; Catch-related constraints > access constraints > interpersonal > intrapersonal	***
Kearney (2006) [38]	U.S.	QUAL/Qualitative description	40	Residents with limited mobility in long-term care	Nearby nature of long-term care facility	Passive and active	Perceived mental and physical health; invigoration; social interaction; life attitude, etc. ↑	Passive contact is more popular among the participants; Plants selection is most popular for the place preference. Quietness, accessibility, resting places and materials are also important. Staff assistance and easy access are helpful for barriers.	**
McAVoy (2006) [39]	U.S.	QUAL/Phenomenology	193 (74 with disabilities and 119 without)	Different kinds of disabilities	Wilderness	Active interaction/Wilderness trips mostly water related	Relaxation, (Self-)awareness, personal growth/challenge, self-confidence; Personal relationship; positive life attitude ↑	A balance between accessible use and enjoyment of wilderness should be achieved; Information about the level of access is useful	****
Motte (2016) [40]	AUS	QUAL/Case study	-	Special need groups, mainly elderly and people with disabilities	Therapeutic gardens in special needs facilities	-	-	Safe and accessible paths; Avoid space confusion; meditation areas; strong seating; shelters for bad weather; active gardening beds	*
Meneghello (2014) [41]	IT	QUAN/Quantitative descriptive studies	28	Multi-kinds (SCI, PD, MS, etc.)	Neurorehabilitation Garden	Active involvement/GT	Self-esteem, relaxation, social involvement ↑	Value being outdoor; GT less fatigue than physiotherapy	**

QUAL-qualitative; QUAN-quantitative; MM-mixed methods; SCI—Spinal cord injury; PD—Parkinson’s disease; MS—multiple sclerosis; FOG-freeze of gait; HRQoL-health related quality of life; OET—Outdoor Experiential Therapy; NW—Nordic Walking. “*”,”**”,”***” and “****” is the scoring system of MMAT. The number of the * indicates the number of criteria met by the evaluated study. There are 4 criteria regarding the quality of methodology for a certain kind of study.

**Table 4:** Summary of the main health benefits of nature-based activities described in the studies.

Items	CAT	Physical Health									Mental Health											Social Health				
**CAT**	**Variables**	**Strength**	**Stamina**	**Pain relief**	**Balance**	**Reaction**	**PHY Independence**	**Perceived PHY H**	**Skill Performance**	**Cardio-respiratory**	**Awareness**	**Renewal**	**Perceived PSY H**	**Mood**	**Cognition**	**Relaxation**	**Stress Reduction**	**Self-Satisfaction**	**Self-esteem**	**Self-confidence**	**Life attitude**	**Relationship**	**Interaction**	**Involvement**	**Willingness**	**Support**
**Passive**	Watch			*													*									
	**Stay in**							*				*	*			*	*						*			
**Active**	WT										*								*	*	*	*				
	Gardening	*						*					*				*						*			
	Skiing						*		*									*	*	*		*				*
	Canoeing								*										*		*	*			*	
	Walking			*	*	*			*	*					*											
	NW			*	*	*			*	*					*											
	Fishing												*			*			*	*			*			
	Kayaking	*	*											*					*	*	*	*		*		*
**Rehabilitative**	OET								*					*					*	*	*			*		
	RT	*			*	*																				

PHY H-physical health; PSY H-psychological health; OET—Outdoor Experiential Therapy; RT—Rehabilitation Training; NW—Nordic Walking; WT—wilderness trip. A “*” in a cell of the table indicates that the activity from that row has the benefit from the column it belongs to.
